# Development of a standardized method of peripherally inserted central catheter (PICC-line) bedside installation

**DOI:** 10.1186/cc13318

**Published:** 2014-03-17

**Authors:** J Hobeika, K Serri, M Albert

**Affiliations:** 1 Hôpital Sacré-Coeur de Montréal, Montreal, Canada

## Introduction

Bedside insertion of peripherally inserted central catheters (PICCs) results in tip malposition in up to 48% [1], with catheters frequently terminating in the internal jugular vein (IJV) or in peripheral veins [2]. In an attempt to reduce tip malposition, we developed a standardized approach to PICC installation. This study aims to validate that method.

## Methods

From a 34-bed adult ICU, we retrospectively reviewed PICC insertions over a 6-month period before the intervention program (control group). We designed a prospective interventional pilot study on 40 consecutive patients (intervention group). Patients in the intervention group were positioned in a standardized fashion and the PICC length was measured from easily identified anatomic landmarks. During PICC insertion, the patient's head was either rotated ipsilaterally to the site of PICC insertion or the ipsilateral IJV was manually compressed, depending on the patient's capacity to collaborate. Once the PICC was inserted, an ultrasound survey was conducted to identify the catheter in the subclavian vein (SC) and ensure its absence in the ipsilateral IJV. The primary endpoint was defined as PICC tip position, obtained from the post-procedural chest X-ray. A catheter was considered to be in an optimal position if the tip resided in the distal third of the superior vena cava (SVC), adequate if it resided between the subclavian vein (SC) and the distal third of the SVC, and aberrant if in any other location.

## Results

In the retrospective control arm, 105 PICCs were reviewed for tip position. Optimal, adequate, and aberrant positions were found in 22 (21%), 49 (47%), and 34 (32%) respectively, in comparison with 17 (43%), 15 (38%), and eight (20%) in the intervention group (*P *< 0.05 between both groups). In the control arm, 11 (10%) PICCs terminated outside the central venous system, whereas none failed to achieve central venous access in the intervention arm.

## Conclusion

Using the standardized method described above, PICC tip positioning can be greatly improved. In our results, 100% of catheters placed using the standardized method allowed for central venous access. This pilot study paves the way for a larger, multicentric evaluation of the bedside installation method of PICC.
